# Molecular mechanism of benzo [a] pyrene regulating lipid metabolism via aryl hydrocarbon receptor

**DOI:** 10.1186/s12944-022-01627-9

**Published:** 2022-01-20

**Authors:** Wei Lou, Meng-di Zhang, Qi Chen, Tu-Ya Bai, Yu-Xia Hu, Feng Gao, Jun Li, Xiao-Li Lv, Qian Zhang, Fu-Hou Chang

**Affiliations:** 1 Department of Pharmacology of Pharmaceutical College, Inner Mongolia Medical University, Hohhot, 010010 China; 2 Department of Pharmacy, Inner Mongolia Autonomous Region Hospital of Traditional Chinese Medicine, Hohhot, 010010 China; 3 Inner Mongolia Research Center for Drug Screening, Inner Mongolia Medical University, Hohhot, 010110 China; 4 The Center for New Drug Safety Evaluation and Research, Inner Mongolia Medical University, Hohhot, 010010 China

**Keywords:** Benzo [a]pyrene, Aryl hydrocarbon receptor, Lipid metabolism, Inflammatory factors

## Abstract

**Background:**

Benzo [a] pyrene (BaP), a potent carcinogen, has been proved that it has toxicological effects via activation the aryl hydrocarbon receptor (AhR) pathway. AhR can participate in regulating lipogenesis and lipolysis. This topic will verify whether BaP regulates lipid metabolism via AhR.

**Methods:**

(1) C57BL/6 mice were gavaged with BaP for 12 weeks to detect serum lipids, glucose tolerance, and insulin resistance. Morphological changes in white adipose tissue (WAT) were detected by Hematoxylin and Eosin staining. The mRNA expression levels of adipogenesis-related factors included recombinant human CCAAT/enhancer binding protein alpha (C/EBPα), peroxisome proliferator-activated receptor gamma (PPARγ), and fatty acid binding protein 4 (FABP_4_) and inflammatory factors included nuclear factor kappa-B (NF-κB), monocyte chemotactic protein-1 (MCP-1), and tumor necrosis factor alpha (TNF-α) were detected using PCR. (2) Neutral lipid content changes in differentiated 3 T3-L1 adipocytes treated with BaP with and w/o AhR inhibitor were detected by Oil red staining. The protein expression levels of adipogenesis- and decomposition-related factors included PPARγ coactivator-1 alpha (PGC-1α), and peroxisome proliferation-activated receptor alpha (PPARα) were detected using western blotting. The mRNA expression levels of inflammatory factors were detected using PCR.

**Results:**

(1) BaP inhibited body weight gain, decreased lipid content, increased lipid levels, and decreased glucose tolerance and insulin tolerance in mice; (2) BaP reduced the expressions of C/EBPα, PPARγ, FABP_4_, PGC-1α, and PPARα and increased the expressions of NF-κB, MCP-1, and TNF-α by activating AhR.

**Conclusion:**

BaP inhibit fat synthesis and oxidation while inducing inflammation by activating AhR, leading to WAT dysfunction and causing metabolic complications.

## Introduction

Benzo [a] pyrene (BaP) is a condensed ring aromatic hydrocarbon containing a benzene ring, which is widely distributed in the environmental medium of human survival, such as food grilling, car exhaust, and cigarette smoke [1]. A large number of epidemiological studies have shown that BaP is closely related to the occurrence of lung cancer, bladder cancer, skin cancer and breast cancer [2–4]. It is highly fat-soluble and therefore tends to accumulate in fat and liver tissues [5]. It is a classic exogenous ligand of Aromatic hydrocarbon receptor (AhR), which can activate AhR to trigger various toxicological effects.

AhR is a ligand-activated transcription factor that can bind a variety of ligands, including exogenous aromatic hydrocarbons and endogenous ligands [6]. In the absence of ligands, it binds to the molecular chaperone and is present in the cytoplasm [7]. After binding to BaP, AhR forms a heterodimer with the aryl receptor nuclear translocator (ARNT) and transports it to the nucleus to initiate downstream gene activation [8]. It can participate in regulating lipid metabolism. Lee et al. [9, 10] found that in AhR transgenic mice, constitutively activated AhR can lead to dyslipidemia by inhibiting mitochondrial fatty acid oxidation. Moreover, the expression of AhR gradually decreases during the differentiation of 3 T3-L1 preadipocytes, indicating that it can negatively regulate the differentiation of preadipocytes [11, 12]. Taken together, it plays an important role in the formation and decomposition of lipids.

Lipid metabolism of WAT includes processes such as lipogenesis and lipolysis [13]. Lipogenesis involves the transformation of precursor adipocytes into mature adipocytes, mediated by peroxisome proliferator-activated receptor gamma (PPARγ), recombinant human CCAAT/enhancer binding protein alpha (C/EBPα), and fatty acid binding protein 4 (FABP4) [14]. Under stimuli, such as hunger and cold, fat mobilization can occur in WAT to promote the decomposition of triglycerides to form glycerol and free fatty acids (FFAs). FFAs activate the PPARγ coactivator-1 alpha (PGC-1α) and peroxisome proliferation-activated receptor alpha (PPARα) to generate ATP [15]. WAT not only regulates energy storage and decomposition but also but also regulates inflammation. Nuclear factor kappa-B (NF-κB), monocyte chemotactic protein 1 (MCP-1), and tumor necrosis factor-α (TNF-α) are typical representatives of inflammatory factors, which can interfere with insulin signal transduction, thereby affecting the lipid metabolism of WAT [16]. Under normal circumstances, lipids are synthesized and decomposed in WAT to maintain a dynamic balance. Abnormalities in storage and excessive decomposition of lipids in WAT cause conditions such as insulin resistance, hyperlipidemia, fatty liver disease, and atherosclerosis.

Our laboratory has been engaged in the research of the BaP-AhR-ARNT pathway for many years, however, there is no report on whether or not BaP affects lipid metabolism. Therefore, the aim of the present study was to elucidate how BaP affects lipid production, decomposition, and inflammatory response by activating AhR, which leads to disorders of lipid metabolism.

## Materials and methods

### Materials

BaP, IBMX, insulin, Dexamethasone was purchased from Sigma (California USA). Triglyceride detection kit, total cholesterol detection kit, low-density cholesterol lipoprotein (LDL-C) detection kit and high-density lipoprotein cholesterol (HDL-C) detection kit were purchased from Zhongsheng Beikong Biotechnology Co., Ltd. (Bejing, China). PPARγ rabbit monoclonal antibody, C/EBPα rabbit monoclonal antibody were purchased from Cell Signaling Technology (Boston, USA). FABP4 rabbit monoclonal antibody, CYP1A1 rabbit monoclonal antibody, PGC1-α goat polyclonal antibody, PPARα rabbit monoclonal antibody were purchased from Abcam Cambridge, UK). RevertTra Ace qPCR RT kit and SYBR Green Realtime PCR Master Mix were purchased from TOYOBO company (Tokyo, Japan) Dulbecco’s Modified Eagle Medium and fetal bovine serum were purchased from Gibco company (Grand Island, NY).

## Methods

### Experimental animals and grouping

After being fed with common feed for 1 week, sixty C57BL/6 mice (license number: SCXK (Beijing) 2016–0002) were randomized equally in five groups: control group, vehicle group, low-, medium-, and high-dose BaP group. The mice in the control group were not treated. The mice in the vehicle group were administered with 0.1 ml corn oil per 10 g body weight every day. Mice in the low-, medium-, and high-dose BaP groups were administered with 0.45, 0.90, and 1.80 mg/kg/day of BaP in corn oil solution, respectively. After 12 weeks of administration, the animal materials and relevant indicators were tested.

### BaP preparation

The BaP powder was dissolved in different concentrations of dimethyl sulfoxide to create a working solution. Subsequently, different concentrations of BaP were added to the working solution, the basal medium, and the induction solution to obtain the final concentrations of 0.1, 1, and 10 μmol/L, respectively, as reported previously [17].

### Cell culture and differentiation

3 T3-L1 preadipocytes were cultured in high-glucose Dulbecco’s modified Eagle’s medium, containing 10% fetal bovine serum and 1% penicillin/streptomycin. Incubation was performed under 5% CO_2_ at 37 °C. The cells adhered to the bottom of the plate and could be used for the following culture when they grew by 80%. The medium was changed every 2–3 days. 3 T3-L1 preadipocytes were grown in the logarithmic phase. They were induced differentiation until the cell density was approximately 100%. Induction solution I containing 0.5 μmol/L of IBMX, 10 mmol/L of dexamethasone, and 10 μg/ml of insulin was added at 37 °C and 5% CO_2_ after culturing in the incubator for 48 h. It was switched to induction solution II containing 10 μg/ml of insulin and cultured for 48 h [18, 19]. Subsequently, it was changed to the basic medium and cultivated for four days, changing the medium every 48 h. Follow-up experiments were conducted. The experiments were repeated thrice.

### Treatment of differentiated 3T3-L1 adipocytes with BaP with and without AhR inhibitor

3 T3-L1 preadipocytes grown in the logarithmic phase were treated with and without 1 μmol/L of the AhR inhibitor CH223191 for 24 h and then with 1 μmol/L of the BaP-inducing solution to induce differentiation. The experiments were repeated thrice.

### Glucose tolerance test (GTT) and insulin tolerance test (ITT)

C57BL/6 mice were fasted for 16 h after modeling for BaP for 12 weeks. Each mouse was intraperitoneally injected with 2 g/kg of the glucose solution. Blood was collected from the tip of the tail to measure fasting blood glucose (0 min) and blood glucose at 15, 30, 60, 90, and 120 min after the glucose injection. C57BL/6 mice were fasted for 16 h the day after the GTT experiment. Each mouse was injected intraperitoneally with 0.5 U/kg of insulin. Blood at the tip of the tail was used to measure fasting blood glucose (0 min) and blood glucose at 15, 30, 60, 90, and 120 min after the glucose injection.

### Histological and biochemical analyses

Part of the epididymal fat tissue was fixed in 4% paraformaldehyde and then dehydrated in paraffin-embedded graded alcohol. Sections of 4 μm were cut out and stained with Hematoxylin and Eosin (HE) for histological examination.

Blood was collected from retro orbital bleeding after anesthesia with 10% chloral hydrate. After standing for 30 min, the lysate was retained. Serum triglyceride, total cholesterol (TC), LDL-C and HDL-C were detected using a kit with triglyceride detection kit, total cholesterol detection kit, LDL-C detection kit and HDL-C detection kit**.**

### Real-time quantitative polymerase chain reaction

The total RNA was extracted using a kit and reverse transcribed to obtain cDNA. Polymerase chain reaction was performed. Analysis of the relative expression of the target gene was based on the 2^-△△CT^ principle. Using the stably expressed gene β-actin as an internal reference, each sample was repeated thrice in parallel, and the average value was analyzed. Table 1 shows primer sequences.

**Table 1:** Primer sequences

Gene	Primer sequence (5′—3′)
C/EBPα	Forward primer 5′-TATAGACATCAGCGCCTACATC-3’
	Reverse primer 5′-TTCTTGTCCACCGACTTATGAC-3’
PPARγ	Forward primer 5′-CCAAGAATACCAAAGTGCGAT-3’
	Reverse primer 5′-TCACAAGCATGAACTCCATAG-3’
FABP_4_	Forward primer 5′-CATCCGGTCAGAGAGTACTTT-3’
	Reverse primer 5′-TAGGGTTATGATGCTCTTCACC-3’
β-Actin	Forward primer 5′-CTACCTCATGAAGATCCTGACC-3’
NF-κB	Reverse primer 5′-CACAGCTTCTCTTTGATGTCAC-3’
	Forward primer 5′-CTGAAAAGCACCTGACAAAAGA-3’
MCP-1	Reverse primer 5′-CTGTGTAGCCATCTGTTGAGTT-3’
	Forward primer 5′- TTTTTGTCACCAAGCTCAAGAG-3’
TNF-α	Reverse primer 5′- TTCTGATCTCATTTGGTTCCGA-3’
	Forward primer 5′-ATGTCTCAGCCTCTTCTCATTC-3’
CYP1A1	Reverse primer 5′-GCTTGTCACTCGAATTTTGAGA-3’
	Forward primer 5′-ACCCTTACAAGTATTTGGTCGT-3’
	Reverse primer 5′-GTCATCATGGTCATAACGTTGG-3’

### Western blotting assay

The cellular or tissues were homogenized in radioimmunoprecipitation assay (RIPA) buffer. Prior to homogenization, protease inhibitor cocktail and phosphatase inhibitor was added. Protein was quantified via Protein Assay Kit. A total of 50 μg protein from each sample was loaded, separated via SDS–PAGE gels, and then transferred to PVDF membranes. After blocking for 2 h, the membrane was incubated with the corresponding antibodies targeting PPARγ (1:1000), C/EBPα (1:1000), FAB_4_ (1:1000), PGC1-α (1:1000), PPARα (1:1000), CYP1A1 (1:500) or β-actin (1:1000) at 4 °C overnight. Subsequently, the membranes were processed with appropriate HRP-conjugated secondary antibodies (1:5000) with regard to the proteins of interest at room temperature for 1 h. The protein levels were analyzed using Image J and normalized relative to that of the internal control β-actin.

### Oil red O staining

The oil red O staining solution was configured and stored in dark. After fixing the treated cells with formalin, the oil red O staining solution was added, stained for 20–30 min in dark, washed with water, and studied under a microscope. To extract the oil red O dye adsorbed by the lipid, 60% isopropyl alcohol was added. Absorbance at 490 nm was measured with a microplate reader.

### Statistical analysis

Graphpad Prism 6.0 was used for statistical analysis and graphing. Differences between measurement data groups were tested by one-way analysis of variance or non-parametric rank sum test. The data were expressed by^−^x ± SEM, and *P* < 0.05 was used as the standard for statistically significant differences.

## Results

### Effects of BaP on body weight, epididymal fat and subcutaneous fat tissue weight, and HE staining in mice

**Fig. 1:**
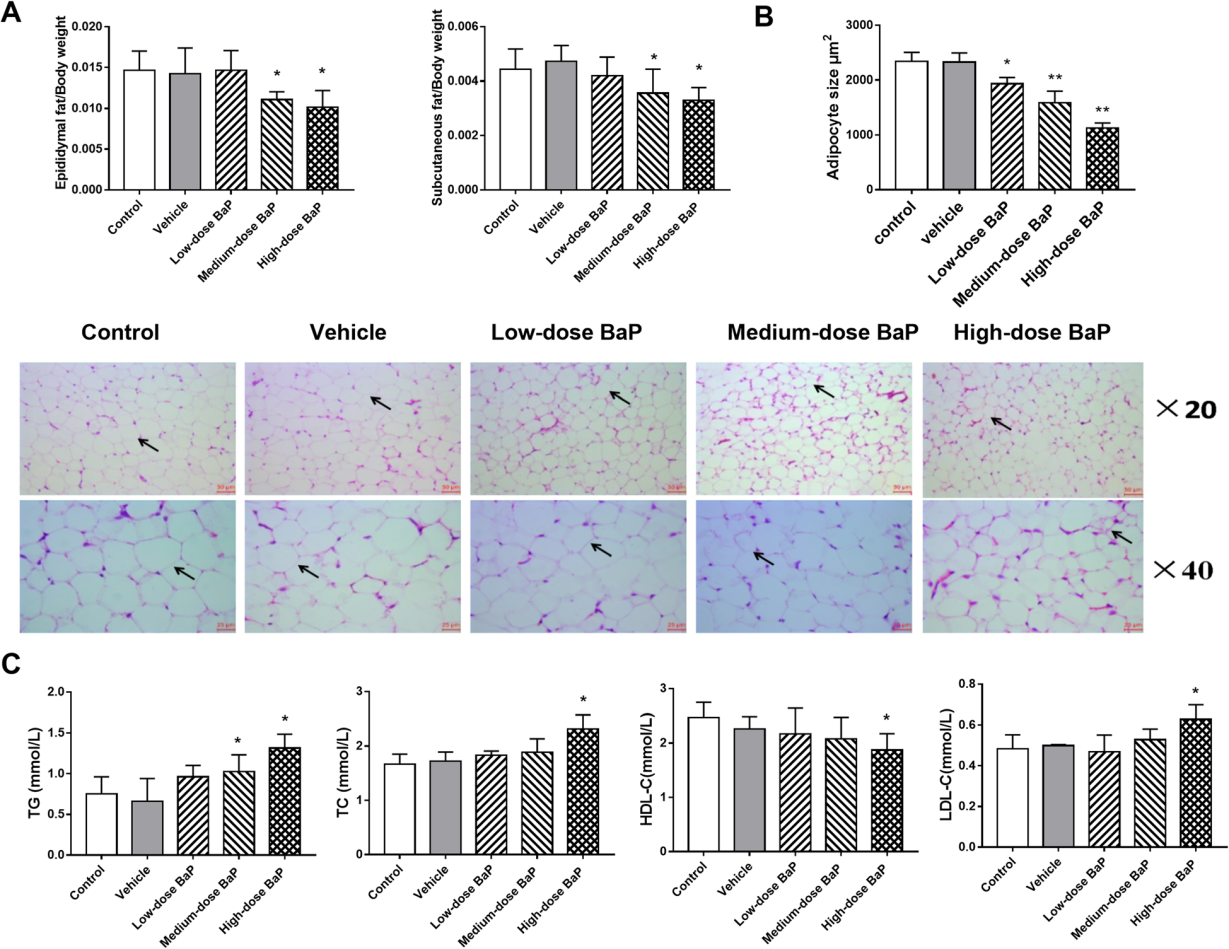
Effect of different concentrations of BaP on mouse epididymal and subcutaneous fat tissue and contents of triglyceride, TC, HDL-C, and LDL-C (**A**: fat tissue weight; **B**: fat cell size; **C**: serum lipids results; compared to the vehicle group: *P < 0.05, **P < 0.01, *n* = 3)

In this experiment, we first observed the effect of intragastric administration of BaP on the body weight of C57BL/6 mice after 12 weeks. The results showed that medium-dose (0.90 mg/kg/day) and high-dose (1.80 mg/kg/day) BaP could slow weight gain in mice (Table 2). As shown in Fig. 1A, compared to the vehicle group, the weight of epididymal and subcutaneous adipose tissue in mice in the medium-dose (0.90 mg/kg/day) and high-dose (1.80 mg/kg/day) BaP groups reduced significantly (*P* < 0.05). The results of HE staining (Fig. 1B) showed that contrary to the vehicle group, each dose level group of BaP depicted suppressing effects on the lipid content of WAT (*P* < 0.05, *P* < 0.01). The aforementioned results indicate that BaP can inhibit WAT expansion and fat deposition.

**Table 2:** BaP gavage C57BL/6 mice weight gain in 12 weeks (^−^*x* ± s, *n* = 12)

time/week	body weight/g				
	control	vehicle	low-dose BaP	medium-dose BaP	high-dose BaP
2	23.70 ± 0.40	23.89 ± 0.83	23.24 ± 0.44	22.95 ± 1.19	23.11 ± 0.99
4	25.10 ± 0.50	25.83 ± 0.60	24.93 ± 0.42	24.87 ± 1.25	24.92 ± 1.23
6	28.60 ± 0.70	29.10 ± 1.01	28.32 ± 1.47	27.78 ± 0.57	27.35 ± 1.77*
8	30.45 ± 0.38	30.90 ± 0.99	30.09 ± 0.68	29.70 ± 0.56*	28.50 ± 1.49*
10	30.78 ± 0.22	31.02 ± 1.26	30.25 ± 0.37	29.78 ± 0.47*	28.98 ± 1.46*
12	31.54 ± 0.21	31.71 ± 1.20	31.39 ± 0.42	30.26 ± 0.48*	29.30 ± 1.67**

^*^P < 0.05, ^**^ *P* < 0.01 vs vehicle

### Effects of BaP on serum lipids, GTT, and ITT in mice

**Fig. 2:**
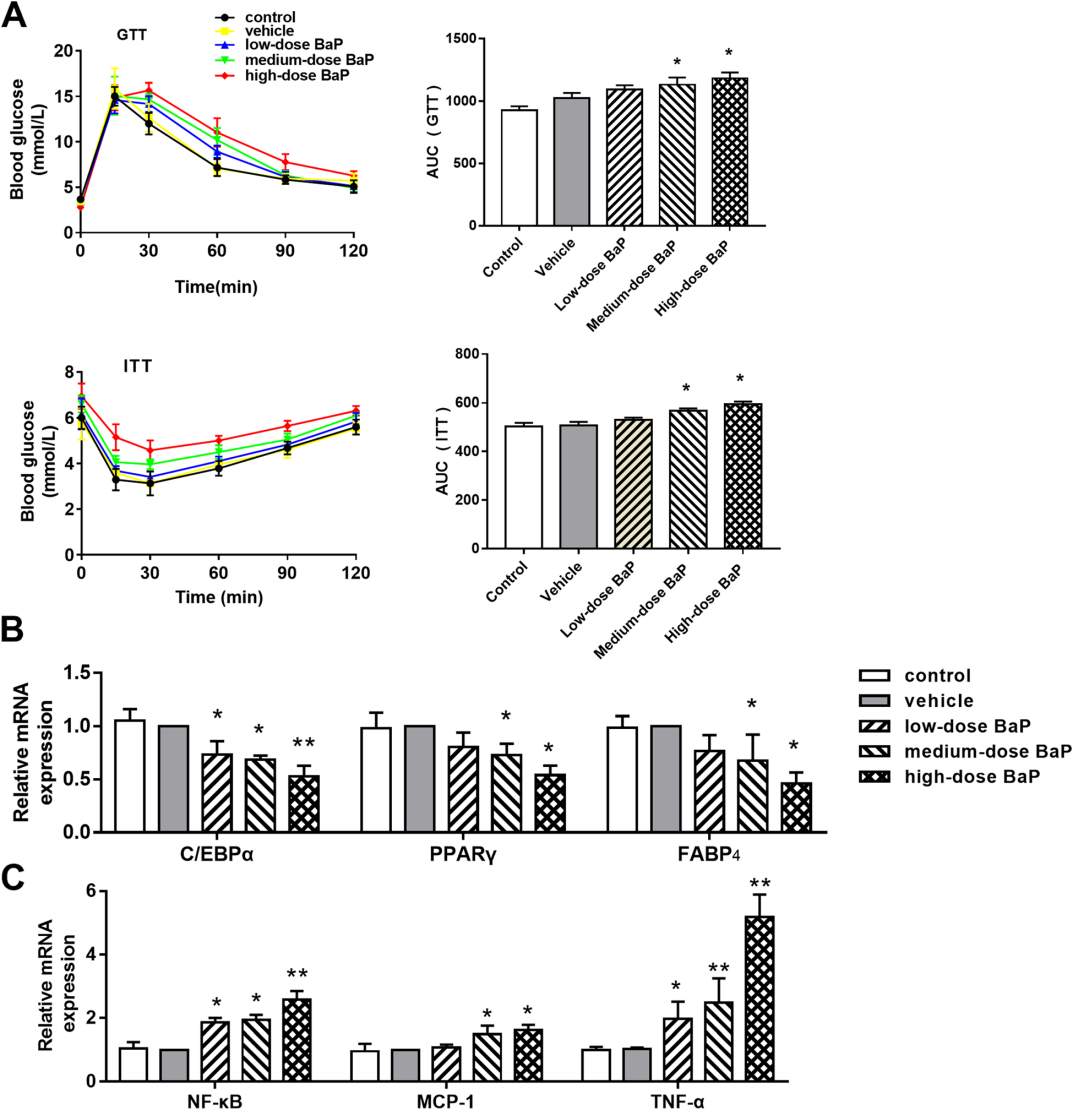
Effects of different concentrations of BaP on GTT, ITT, lipogenesis factors, and inflammatory factors in mouse WAT (**A**: GTT and ITT results; **B**: lipogenesis factor; **C**: inflammatory factor; compared to the vehicle group: * P < 0.05, ** P < 0.01, *n* = 3)

The results of serum lipids showed that medium-dose BaP (0.90 mg/kg/day) increased triglyceride levels (*P* < 0.05), while high-dose BaP (1.80 mg/kg/d) increased triglyceride, TC, and LDL-c levels and decreased HDL-c levels (*P* < 0.05). The aforementioned results indicate that the stimulation of medium- (0.90 mg/kg/day) or high-dose BaP (1.80 mg/kg/day) can cause serum lipids disbalance in mice (Fig. 1C). GTT and ITT results show that medium-dose (0.90 mg/kg/day) and high-dose BaP (1.80 mg/kg/day) can interfere with the normal glucose metabolism of mice and reduce the body’s sensitivity to insulin (Fig. 2A).

### Effects of BaP on lipogenesis and inflammatory factors in WAT of mice

Compared to the vehicle group, mRNA expression of C/EBPα were reduced by 26.6, 31.7 and 47.3% respectively in low-, medium, and high-dose BaP groups (*P* < 0.05). Compared to the vehicle group, mRNA expression of PPARγ were reduced by 27.1 and 46%, mRNA expression of FABP_4_ were reduced by 32.5 and 53.9% in medium- and high-dose BaP group (*P* < 0.05; Fig. 2B).

Compared to the vehicle group, mRNA expression of NF-κB was increased by 85.5, 93.1 and 157%, mRNA expression of TNF-α was increased by 97.3, 148.6 and 417.9% respectively in low-, medium, and high-dose BaP groups (*P* < 0.05), mRNA expression of MCP-1 was increased by 66 and 66.3% respectively in medium, and high-dose BaP groups (*P* < 0.05). The results indicate that BaP may interfere with lipid metabolism by inhibiting lipogenesis and increasing the expression of inflammatory factors.

### Effects of different concentrations of BaP on neutral lipid metabolism and neutral lipid content of 3 T3-L1 adipocytes

**Fig. 3:**
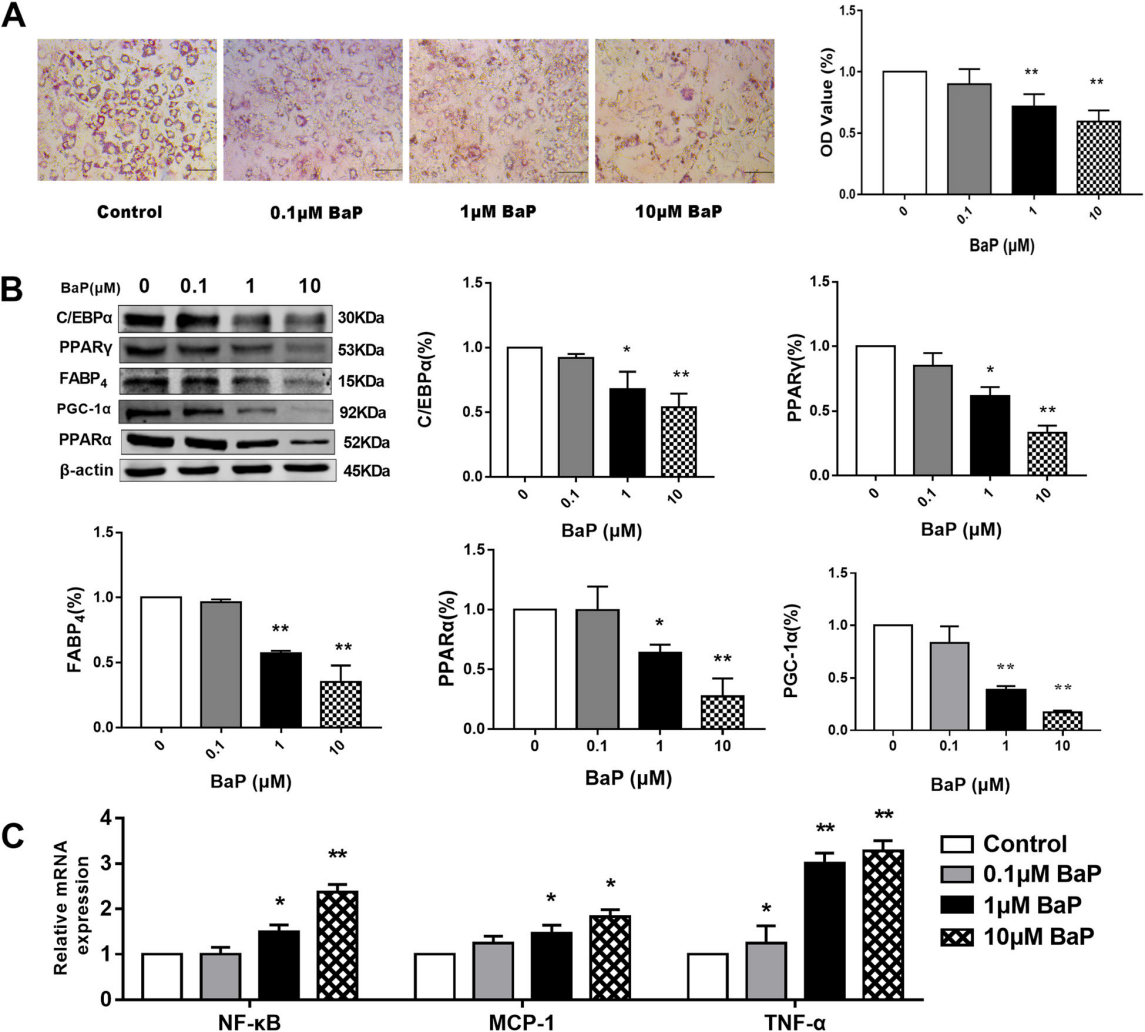
Effect of different concentrations of BaP on neutral lipid content and the expression of lipid metabolism-related factors and inflammatory factors in 3 T3-L1 cells (**A**: oil red O staining; **B**: lipid metabolism-related factors; **C**: inflammatory factors; compared to the control group: * P < 0.05, ** P < 0.01, *n* = 3)

Compared to the control group, neutral the lipid content of 3 T3-L1 adipocytes were reduced by 9.92% (*P* > 0.05), 28.08% (*P* < 0.01), and 40.42% (*P* < 0.01) respectively after treatment with 0.1, 1 and 10 μmol/L of BaP (*P* < 0.01) (Fig. 3A,). The results show that BaP can inhibit neutral lipid accumulation in 3 T3-L1 adipocytes.

### Effects of BaP on the expression of lipid metabolism-related factors and inflammatory factors in 3 T3-L1 adipocytes

Compared to the control group, protein levels of C/EBPα were significantly reduced by 32.01 and 45.92% after the administration of 1 or 10 μmol/L of BaP, protein levels of PPARγ were significantly reduced by 38.09 and 66.74% after the administration of 1 or 10 μmol/L of BaP (*P* < 0.05) (Fig. 3B), protein levels of FABP_4_ were significantly reduced by 42.79 and 64.93% after the administration of 1 or 10 μmol/L of BaP (*P* < 0.05) (Fig. 3B). The aforementioned results show that BaP can inhibit the expressions of C/EBPα, PPARγ, and FABP_4_ (*P* < 0.05), thereby inhibiting the transformation of pre-adipocytes into mature adipocytes and reducing the lipid content, consistent with the in-vivo experimental results.

Under normal circumstances, the synthesis and decomposition of lipids maintain a dynamic balance. When fat decomposition occurs abnormally, it affects fat metabolism [20]. Therefore, we tested the effect of BaP on the fatty acid transfer in-vitro (Fig. 3B). Compared to the control group, the protein expressions of PPARα decreased by 36.31 and 72.55% after the administration of 1 or 10 μmol/L of BaP, the protein expressions of PGC-1α decreased by 61.88 and 82.94% after the administration of 1 or 10 μmol/L of BaP (*P* < 0.05). The results show that BaP can inhibit fatty acid transfer related factors.

Compared to the control group, the mRNA expressions of NF-κB was increased by 48.7 and 136.9% in 1 μmol/L, and 10 μmol/L of BaP group, respectively; the mRNA expressions of MCP-1 was increased by 45.9 and 82.2% in 1 μmol/L, and 10 μmol/L of BaP group, respectively; the mRNA expressions of TNF-α was increased by 34.9, 200.8, and 227.6% in 0.1 μmol/L, 1 μmol/L, and 10 μmol/L of BaP group, respectively. The results show that BaP stimulation at the cellular level can induce the expression of inflammatory factors, consistent with the results of in-vivo experiments.

### BaP interfering with lipid metabolism and affecting the CYP1A1 expression via AhR

**Fig. 4:**
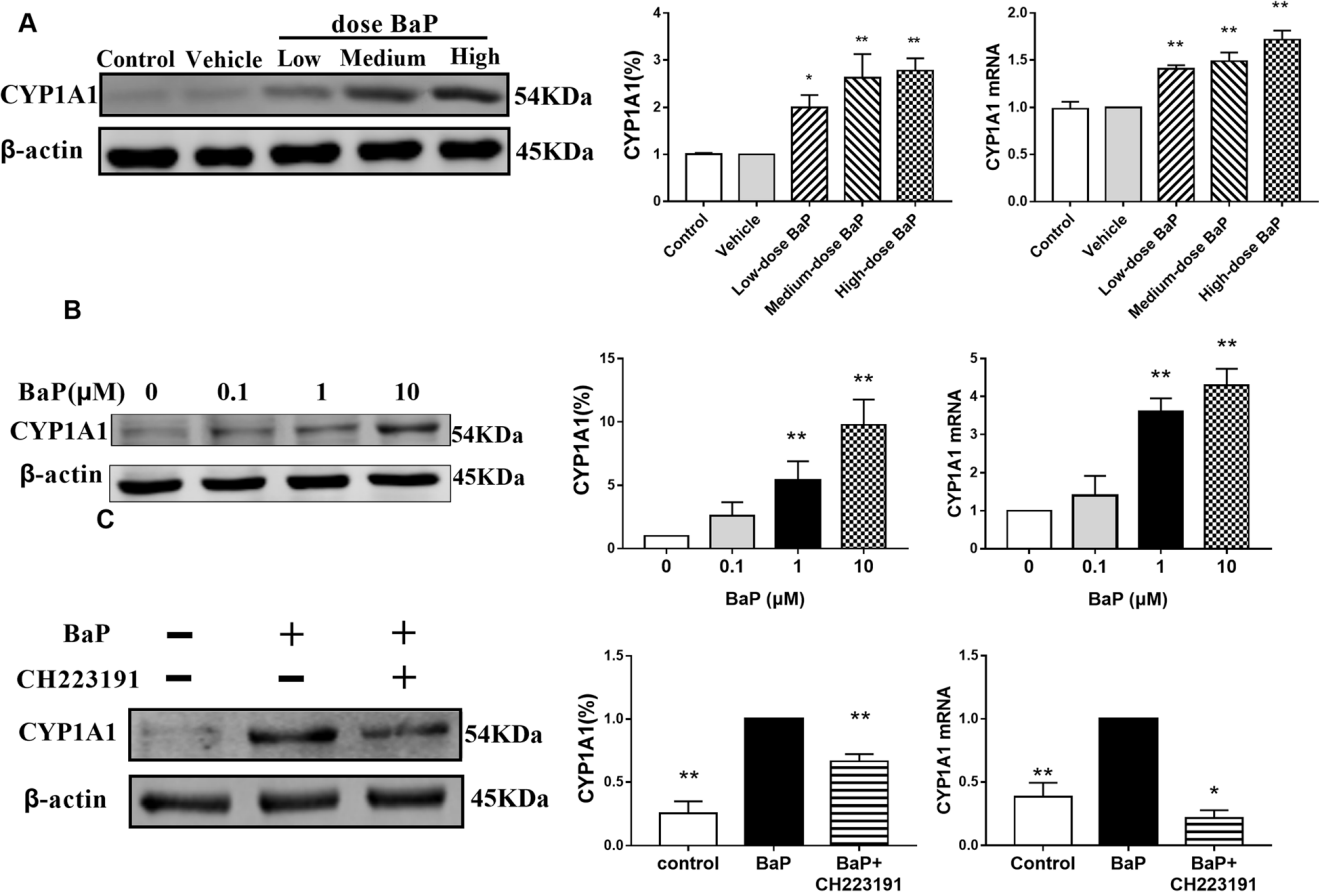
Effect of BaP on mRNA and protein expressions of CYP1A1 (**A**: mouse; **B**: 3 T3-L1 cells; **C**: adding AhR inhibitor. * P < 0.05, ** P < 0.01, *n* = 3)

In-vivo and in-vitro experiments showed that compared to the control group, the BaP group had significantly increased mRNA and protein expressions of CYP1A1 (*P* < 0.05; Fig. 4A and B). After 1 μmol/L of the AhR inhibitor CH223191 treated 3 T3-L1 adipocytes for 24 h, the effect of BaP on CYP1A1 mRNA and protein levels reduced significantly (*P* < 0.05; Fig. 4C). Therefore, 1 μmol/L of CH223191 and 1 μmol/L of BaP were used for subsequent experiments.

### Effects of BaP on the expression of lipid metabolism-related factors and inflammatory factors in 3 T3-L1 adipocytes in the presence of AhR inhibitors

**Fig. 5:**
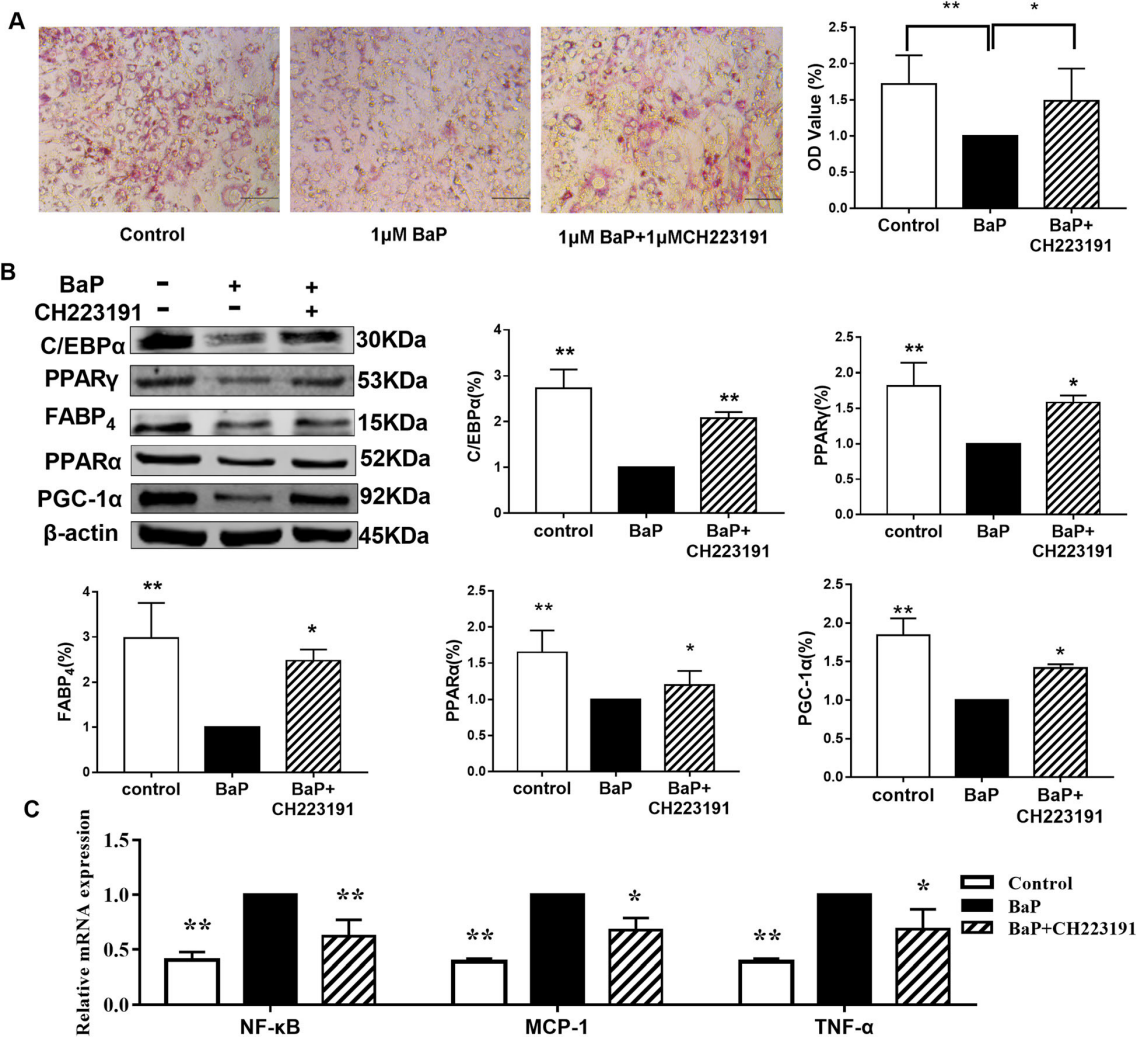
Effect of BaP on neutral lipid content and the expression of lipid metabolism-related factors and inflammatory factors in 3 T3-L1 cells in the presence of AhR inhibitors. (**A**: oil red O staining; **B**: lipid metabolism-related factors; **C**: inflammatory factors; compared to the BaP group: * P < 0.05, ** P < 0.01, *n* = 3)

Compared to the BaP group, the lipid content of 3 T3-L1 adipocytes in the BaP + CH223191 group increased by 53.9% (*P* < 0.05; Fig. 5A). Compared to the BaP group, the expressions of C/EBPα, PPARγ, FABP_4_, PGC-1α, and PPARα in the BaP + CH223191 group increased by 108.3, 58.3, 147.1, 41.4, 20% respectively.

Compared to the BaP group, the mRNA expression levels of NF-κB, MCP-1, and TNF-α in the BaP + CH223191 group reduced by 40.5, 38.9 and 39%. After inhibiting AhR, the effects of BaP on C/EBPα, PPARγ, FABP_4_, PGC-1α, PPARα, NF-κB, MCP-1, and TNF-α were weakened. Thus, BaP may inhibit lipogenesis and fatty acid decomposition and simultaneously promote the expression of inflammatory factors by activating AhR (Fig. 5C).

## Discussion

Polycyclic aromatic hydrocarbon compounds are a class of ubiquitous pollutants. However, studies on the toxicological effects of BaP, another representative of polycyclic aromatic hydrocarbons, have been overlooked. BaP is a harmful substance that often exposed in environmental pollution in daily life. Its effect on lipid metabolism and mechanism of action should be clarified to prevent metabolic disease.

This experiment found that BaP can inhibit weight gain and reduce the weight of WAT. The results of serum lipids testing revealed that BaP can increase triglyceride, TC, and LDL-C in the serum of mice and reduce HDL-C, which in turn causes imbalance in serum lipids. The results of GTT and ITT show that BaP can render mice glucose metabolism abnormal and reduce insulin sensitivity. Thus, BaP can cause lipid metabolism disorders in experimental animals.

WAT performs the function of storing energy and participating in lipid metabolism [21]. When the energy balance of the body changes, WAT expands cell size and number to increase lipid storage [22]. This process is called adipose tissue remodeling. PPARγ, C/EBPα, FABP_4_ are the main regulators in the process of adipogenesis, and they play an important role in lipid synthesis and glucose homeostasis [23–25]. The in-vivo and in-vitro results of this experiment showed that BaP can inhibit PPARγ, C/EBPα, and FABP_4_, thereby inhibiting WAT expansion. When WAT cannot properly expand to store energy, the extra energy intake is converted into lipid ectopic deposition in other tissues, leading to lipotoxicity including local inflammation and insulin resistance [26]. This experiment revealed that BaP caused dyslipidemia and abnormal glucose metabolism, and it might be related to the inhibition of WAT expansion and lipid deposition.

Mitochondrial biogenesis is a process that increases the quality and quantity of mitochondria in cells. Mitochondrial biogenesis and remodeling in WAT are associated with fatty acid uptake and oxidation [27]. Elevated PGC-1α promotes the translocation of mitochondrial termination factor A into the mitochondria, and mitochondrial biogenesis. Activation of the downstream gene PPARα of PGC-1α can also enhance the β-oxidation of peroxisome fatty acids, thereby reducing lipid levels, including TC and triglyceride [28–30]. Therefore, BaP can inhibit mitochondrial function and is also a molecular mechanism that causes WAT dysfunction.

Macrophage infiltration and accumulation in WAT are related to increased secretion of inflammatory factors. This can affect systemic insulin sensitivity and glucose metabolism as well as participate in inhibiting fat production [31]. It can promote the expression of inflammatory factors by activating signal pathways, such as HIF-1 and STAT-3 [32, 33]. Our study demonstrates that BaP can promote the expression of inflammatory factors NF-κB, MCP-1, and TNF-α. TNF-α can inhibit the transcription of insulin receptor substrate 1, glucose transporter 4, and PPARγ, interfering with insulin receptor signaling in adipose tissue [34]. Insulin resistance can promote the occurrence of type 2 diabetes, cardiovascular disease and dyslipidemia [35]. Kakali G al. foud that the significantly down-regulated expression of adiponectin and its receptor in adipose tissue are related to diabetic dyslipidemia [36]. Inflammatory factor TNF-α and adiponectin regulate each other’s expression in adipocytes [37]. The representative compound of polycyclic aromatic hydrocarbons, TCDD, induces insulin resistance by inducing TNF-α to reduce the expression of adiponectin in C3H10T1/2 adipocytes [38]. Combining the results of GTT and ITT experiments, BaP may decrease insulin sensitivity in mice by inducing the WAT inflammatory response to interfere with the conduction of the insulin signal pathway.

BaP is a classic exogenous ligand of AhR. In the absence of ligands, AhR and molecular chaperones HSP90, p23, and XAP2 form inactive complexes and exist in the cytoplasm [39]. When BaP and AhR are combined, AhR and ARNT form a dimer, which is transferred to the nucleus and induces the transcription of genes containing xenobiotic response element sequences in their promoters, a classic activator in the CYP family [40]. Therefore, this study was not aimed directly at detecting the expression level of AhR but at observing whether or not BaP activates the function of AhR by detecting the expression of CYP1A1. AhR is involved in regulating lipid production and breakdown. Shimba S et al. [41] found that the expression level of AhR during the differentiation of 3 T3-L1 preadipocytes gradually decreased, indicating that AhR may be involved in the process of adipocyte fat synthesis. AhR knockout mice exhibit steatosis in the liver [42, 43]. These results indicate that AhR regulate adipogenesis in various ways. Lee et al. [9] found that constitutive activation of AhR in AhR transgenic mice can lead to disorders of liver lipid metabolism. The mechanism is due to the inhibition of PPARα and then the inhibition of liver fatty acid transfer [44]. Whether BaP, as the exogenous ligand of AhR, regulates adipocyte lipogenesis and free fatty acid breakdown by activating AhR was not shown until our study. Here we demonstrate that AhR inhibition with CH233191 diminishes the effect of BaP on adipogenesis, mitochondrial free fatty acid transfer and inflammatory factors. Therefore, BaP inhibits lipogenesis and free fatty acid decomposition by activating AhR and interferes with lipid metabolism. BaP activates AhR and induces the expression of inflammatory factors, which may affect the transduction of the insulin signaling pathway.

### Comparisons with other studies and what the current work adds to existing knowledge

Past studies have shown that AhR plays an important role in adipocyte differentiation [39, 40] and liver fat metabolism [9, 41, 42]. As an exogenous ligand of AhR, BaP exerts toxicological effects by activating the function of AhR. However, there are few studies on the effect of BaP activating AhR on lipid metabolism. Compared with previous studies, this study first proved that BaP, a polycyclic aromatic hydrocarbon compound, interferes with fat synthesis, causing pathological weight loss in experimental animals, and at the same time disturbing blood glucose and serum lipids. Then it was verified in-vitro that BaP affects lipid metabolism by activating AhR. We propose that AhR constitutes a valuable target to protect metabolic-related diseases caused by environmental pollutants polycyclic aromatic hydrocarbons.

### Strengths and limitations

This study encompassed the effects of BaP on lipogenesis, decomposition, inflammation, and a series of metabolic disorders caused by AhR in in-vivo and external settings. Previous studies have focused on disorders of lipid metabolism caused by obesity. The environmental pollutant BaP causing metabolic disorders involves the dysfunction of adipose tissue as the core mechanism of metabolic complications, regardless of the presence/absence of obesity. Many harmful effects are related to the unhealthy expansion of WAT caused by AhR, including inflammation, changes in adipokines secretion, and mitochondrial dysfunction. Each of them may be a target for the treatment of metabolic diseases caused by the environmental pollutant BaP. However, this study has some limitations. First, due to experimental conditions and time constraints, there is no silencing of AhR to observe the effect of BaP on lipid metabolism. Second, after adding the AhR inhibitor CH223191, the expression level of AhR is not directly measured, but by measuring the content of CYP1A1 to observe whether AhR is inhibited. In the future, we will use AhR knockout technology to directly measure the expression of AhR to further verify the important role of AhR in lipid metabolism.

## Conclusions

Metabolic diseases caused by BaP are related to the unhealthy expansion of WAT caused by AhR, including increaseing inflammatory factors, inhibiting fat formation and FFA transport. Each of them may be a target for the treatment of metabolic diseases caused by the environmental pollutant BaP.

## Data Availability

Not applicable.
